# Multi-tissue transcriptomic study reveals the main role of liver in the chicken adaptive response to a switch in dietary energy source through the transcriptional regulation of lipogenesis

**DOI:** 10.1186/s12864-018-4520-5

**Published:** 2018-03-07

**Authors:** C. Desert, E. Baéza, M. Aite, M. Boutin, A. Le Cam, J. Montfort, M. Houee-Bigot, Y. Blum, P. F. Roux, C. Hennequet-Antier, C. Berri, S. Metayer-Coustard, A. Collin, S. Allais, E. Le Bihan, D. Causeur, F. Gondret, M. J. Duclos, S. Lagarrigue

**Affiliations:** 1 Pegase, INRA, Agrocampus Ouest, 35042 Rennes, France; 2 Ura, INRA, 37380 Nouzilly, France; 3 LPGP, INRA, 35000 Rennes, France; 4 UMR6625, Agrocampus Ouest, IRMAR, 35000 Rennes, France; 5 Current address: Programme Cartes d’Identité des Tumeurs (CIT), Ligue Nationale Contre Le Cancer, 75013 Paris, France; 6 Current address, Institut Pasteur, INSERM U933, 75015 Paris, France

**Keywords:** Chicken, Lipid, Adaptation, Fat diet, Gene expression, Regulation, PUFA, SREBF1, TADA2A

## Abstract

**Background:**

Because the cost of cereals is unstable and represents a large part of production charges for meat-type chicken, there is an urge to formulate alternative diets from more cost-effective feedstuff. We have recently shown that meat-type chicken source is prone to adapt to dietary starch substitution with fat and fiber. The aim of this study was to better understand the molecular mechanisms of this adaptation to changes in dietary energy sources through the fine characterization of transcriptomic changes occurring in three major metabolic tissues – liver, adipose tissue and muscle – as well as in circulating blood cells.

**Results:**

We revealed the fine-tuned regulation of many hepatic genes encoding key enzymes driving glycogenesis and de novo fatty acid synthesis pathways and of some genes participating in oxidation. Among the genes expressed upon consumption of a high-fat, high-fiber diet, we highlighted CPT1A, which encodes a key enzyme in the regulation of fatty acid oxidation. Conversely, the repression of lipogenic genes by the high-fat diet was clearly associated with the down-regulation of SREBF1 transcripts but was not associated with the transcript regulation of MLXIPL and NR1H3, which are both transcription factors. This result suggests a pivotal role for SREBF1 in lipogenesis regulation in response to a decrease in dietary starch and an increase in dietary PUFA. Other prospective regulators of de novo hepatic lipogenesis were suggested, such as PPARD, JUN, TADA2A and KAT2B*,* the last two genes belonging to the lysine acetyl transferase (KAT) complex family regulating histone and non-histone protein acetylation. Hepatic glycogenic genes were also down-regulated in chickens fed a high-fat, high-fiber diet compared to those in chickens fed a starch-based diet. No significant dietary-associated variations in gene expression profiles was observed in the other studied tissues, suggesting that the liver mainly contributed to the adaptation of birds to changes in energy source and nutrients in their diets, at least at the transcriptional level. Moreover, we showed that PUFA deposition observed in the different tissues may not rely on transcriptional changes.

**Conclusion:**

We showed the major role of the liver, at the gene expression level, in the adaptive response of chicken to dietary starch substitution with fat and fiber.

**Electronic supplementary material:**

The online version of this article (10.1186/s12864-018-4520-5) contains supplementary material, which is available to authorized users.

## Background

Feed costs represent more than 60% of charges in meat production from non-ruminant species such as chicken. It is therefore of interest to identify nutritional strategies that could reduce production costs while maintaining production performances. Because cereal costs have increased dramatically over the past 2 years, alternative diets with fiber-rich co-products show a valuable perspective from an economic point of view. To sustain production traits such as growth or breast muscle weight, dietary energy primarily derived from starch in cereal-based diets must be maintained by adding fat sources such as vegetable oils to fiber-rich diets. This results in changing both energy sources and nutrients in diets. To date, different studies have investigated the effects of dietary energy sources, particularly the impact of carbohydrate substitution by fat on growth performance and body composition in meat-type chicken. Plavnik et al. [1] analyzed the effects of using fat vs. carbohydrates (starch grains) in the diets of 7- to 49-day-old broiler chickens. Adrizal et al. [2] compared the effects of a diet based on defatted rice bran supplemented with fat vs. a conventional corn soybean diet in broilers from 4 to 35 days of age. Recently, we evaluated the effects of two diets with either high-lipid, high-fiber content (HF diet) or high-starch content (LF diet) in 22- to 63-day-old broiler chickens [3]. In this study, cellulose – a compound resistant to digestion in the small intestine – was used as an insoluble fiber source in the HF diet, as it was considered a simple diluent of energy. In these three studies, no effect of diets was observed on production performance (i.e.*,* weight gain, feed efficiency or body composition), showing that chicken is prone to adapt to variations in dietary energy sources. This differs from the results obtained in other non-ruminant species, such as pigs, where marked decreases in feed ingestion, weight gain and body fat content have been observed with high-fat, high-fiber diets [4]. However, the molecular mechanisms associated with chicken adaptation to changes in energy source and nutrients have never been investigated. In this context, the present study aimed to evaluate the molecular responses of tissues involved in energy homeostasis to dietary energy sources in chickens by using a transcriptomic approach and proposing key regulators of metabolic pathways. Assuming the involvement of lipid metabolism – one of the keystones underlying energy homeostasis – in this adaptation process, we also compared responses between two broiler lines divergent for abdominal fat content for evaluating an eventual interaction between diet and genotype on gene expression. The three tissues investigated were i) the liver, the key lipogenic organ in birds, which is also involved in many other physiological processes such as oxidation, secretion and detoxification; ii) the white adipose tissue, which is critical for fatty acid storage; and iii) the *Pectoralis* major muscle, one of the most energy-consuming tissues considering its mass. The peripheral blood mononuclear cells (PBMCs) were also analyzed since many studies have now highlighted their relevance to understanding body energy homeostasis, metabolic disease and immunity [5–7]. Because chicken fills a large evolutionary gap between sauropsids and mammals, the present study also aimed to provide new insights into the conservation of the regulatory networks involved in lipid homeostasis. We showed that the main metabolism impacted by changes in dietary energy sources was fatty acid (FA) metabolism, particularly FA synthesis and polyunsaturated FA (PUFA) deposition in the liver. The present study therefore provided a physiological model to provide a better understanding of the regulation of lipogenic gene expression by dietary FA in chickens and the involved transcription factors. We highlighted known and potential regulator genes for this metabolism by combining differential expression, co-expression and genomics co-localization analyses.

## Methods

### Animals and diets

A total of 64 broiler males from two experimental lines (32 per line) divergently selected for abdominal fat content (fat and lean lines, [8] was obtained from the “Pôle Expérimental Avicole de Tours” (INRA, Nouzilly, France). A total of 2 × 16 chickens per line were grown in individual cages and fed the two experimental diets from 21 to 63 days (d) of age. To limit genetic variation, two full sibs from a given family were assigned in one of the two dietary groups. These two diets were isocaloric (12.54 MJ ME/kg) and isonitrogenous (190 g CP/kg) but exhibited either a high-starch and low-fiber low-lipid contents (LF diet) or a low-starch, high-fiber and high-lipid contents (HF diet). Starch derived from wheat seeds in the LF diet considered as the standard diet was partially replaced by rapeseed and soybean oils in the HF diet, and cellulose (insoluble fiber) was included to dilute dietary energy in this specific diet. Consequently, diets showed large variations in starch (51% vs. 38%, for LF and HF diets, respectively), fat (2% vs. 8%), and cellulose (2.1% vs. 6.4%) contents. The amounts of saturated, mono-unsaturated and poly-unsaturated fatty acids were similar in the two diets. Detailed compositions of LF and HF diets are precisely described in our previous study [3] and are summarized in the Additional file 1.

### Tissue sampling

At 63 days of age, 12 chickens per line and per diet were selected for slaughtering: only animals with a body weight close to the average weight of their group were considered to limit inter-individual variability. Chickens were killed 3 h after the last meal intake by decapitation and bleeding. Chickens were previously anaesthetized by bi-temporal electronarcosis. Right after slaughter, liver, *Pectoralis major* muscle and abdominal fat were sampled, snap frozen in liquid nitrogen and stored at − 80 °C until analyses. To prepare peripheral blood mononuclear cells (PBMC), whole blood was collected in the occipital sinus in EDTA tubes. Two mL were centrifuged for 10 min at 2000 g, 4 °C and plasma aliquots were stored at − 20 °C before defining plasmatic parameters. Two additional mL were diluted with an equal volume of 1X PBS, mixed by pipetting and deposited on 3 mL Ficoll (Histopaque 1077, Sigma-Aldrich, Saint-Quentin Fallavier, France). We then proceeded to continuous density gradient centrifugation at 720 g for 10 min without brake. The interphase was collected and washed twice with PBS and PBMC pellet was finally snap frozen in liquid nitrogen and kept at − 80 °C.

### Metabolites and traits

Seven traits related to growth performance and body composition were recorded for the 48 birds: body weight (g), average daily gain (g), average daily feed intake (g/day/bird), *Pectoralis major* muscle weight (g), abdominal fat weight (g), and liver weight (g). We further measured lipid content in liver, adipose tissue and *Pectoralis major* muscle – data are expressed in % (g / 100 g tissue) according to [9]. VLDL, LDL and HDL lipoproteins were determined in plasma according to [10]. Glucose, glycogen and lactate levels were measured in liver following the procedure described in Dalrymple and Hamm [11]. Total cholesterol (mg/L), phospholipids (mg/mL), triglycerides (mg/L) and FA composition (C14:0, C16:0, C16:1, C18:0, C18:1, C18:2, C18:3, C20:0, C20:1, C20:4, C20:5, C22:5, C22:6, % of total lipids) were assessed in liver according to methods described in Chartrin et al. [9]. We finally used these elementary variables to compute the relative percentages in SFA (saturated FA), MFA (mono-unsaturated FA) and PUFA (poly-unsaturated FA), n-6 and n-3 FA families, as well as the n-6/n-3 ratio. For additional information about methods and measures, see [3, 10]. A Student t test was performed using the t.test function in R for testing the dietary effect in each of the three FA classes.

### RNA isolation

Liver (30 mg), muscle (30 mg), adipose tissue (100 mg) samples and PBMC pellet were homogenized in TRIzol (Invitrogen, California, USA). Total RNA was then extracted according to manufacturer’s instructions, re-suspended in 50 μL of RNase-free water and stored at − 80 °C. Total RNA was quantified on a NanoDrop® ND-1000 spectrophotometer (Thermo Scientific, Illkirch, France). Absorbance ratios A260/280 and A260/230 were over 1.7 for all samples. RNA preparations were finally quality checked on an Agilent 2100 Bioanalyzer (Agilent Technologies France, Massy, France). Average RNA integrity numbers were 9.4 ± 0.5 for liver, 8.4 ± 0.5 for muscle, 8.0 ± 0.6 for abdominal tissue, 8.8 ± 0.5 for PBMC.

### Gene expression microarray: Data acquisition

The Agilent custom 8 × 60 K chicken gene expression microarray (GPL19630 for GEO database, and ID042004 for Agilent database) used in this study contained the 43,553 probes from the Agilent commercial 44 K collection (ID 026441, Agilent Technologies France, Massy, France) supplemented with 756 probes genes annotated with GO terms related to lipid metabolism, 5821 additional probes known to be expressed in chicken muscle, adipose tissue and liver according to previous experiments [12] and 955 additional probes corresponding to genes not represented in the standard Agilent collection. All these probes corresponded to 16,736 genes referenced in the chicken Ensembl V70 annotation (http://www.ensembl.org/index.html): 50% of the genes were represented by at least 4 probes, whereas 4464 genes were represented by only 1 probe. The human orthologs have been systematically identified according to the “one-to-one” criteria defined by the Ensembl consortium: 69% out of the 16,736 chicken genes have a one-to-one ortholog, allowing us to retrieve for these genes a human HGNC gene symbol from which we could extract more Gene Ontology (GO) annotations.

Total RNA (150 ng per sample) was labelled with Cy3 dye using the Low Input Quick Amp Labeling kit (Agilent Technologies) following the manufacturer’s instructions. Cy3-labeled cRNA samples were purified, fragmented, and hybridized onto Agilent custom microarray at 65 °C for 17 h using Agilent’s Gene Expression Hybridization Kit. After washing 2 × 1 min at room temperature and then at 37 °C, microarrays were scanned using the Agilent DNA Microarray Scanner G2505C, and images were processed with Agilent Feature Extraction Software (Version 10.7.3.1). Finally, 48, 46, 48 and 44 arrays for liver, adipose, muscle and PBMC respectively were available after hybridization and scan.

### Gene expression microarray: data analysis

All analyses were performed using the R software version 3.1.0. Expression dataset were filtered according to different criteria provided by Agilent: two criteria related to spot quality (gIsManualFlag = 0 & gIsFeatNonUnifOL = 0) and one criterion related to spot fluorescence (gIsWellAboveBG = 1). For the 44 to 48 microarrays available per tissue, the mean percentage of spots discarded according to these standards was lower than 0.5% per microarray, suggesting that technical procedures, from slide production to labelling and hybridization, were successful. A gene was then considered as expressed in one given tissue if at least 80% of its related spots in at least one condition satisfied the three criteria defined above.

Intensities of remaining spots were finally log2 transformed. An in-depth quality-check was conducted on raw data to identify potential outlier by using principal component analysis (PCA) (Additional file 2) and analysis of background and signal intensity variation. This step allowed us to point out some outlier arrays which have been further discarded: 2, 1, 3 and 2 outlier arrays were identified out for liver, adipose, muscle and PBMC respectively. We finally analyzed 178 microarrays: 46, 45, 45 and 42 for liver, adipose, muscle and PBMC respectively. Data were normalized by median centering by array and analyzed by using a two-way analysis of variance (with the R function: Anova (lm())) with line, diet and interaction between line and diet as main effects. An analysis using Limma package [13] provided similar results as Anova. The row *p*-values for each factor (line, diet, line x diet) were adjusted following the Benjamini and Hochberg (BH) multiple testing correction method [14] to control the False Discovery Rate. An adjusted *p*-value < 0.05 threshold and an absolute fold-change > = 1.2 cut-off between conditions were considered as a heuristic way to retain robust differentially expressed probes (DEP) and associated differentially expressed genes (DEG).

### BioMark™ real-time PCR analysis

Total RNA isolated from the 48 chickens was reverse-transcribed using High-Capacity cDNA Reverse Transcription kit (Applied Biosystems, Foster City, CA) following manufacturer’s instructions. cDNAs was diluted 1:20 and subjected to a Specific Target Amplification step using PreAmp Master Mix kit (Fluidigm Corporation) with a mixture of all primer pairs and 14 cycles of pre-amplification. The BioMark™ 96.96 Dynamic Array (Fluidigm Corporation) for real-time qPCR was used to simultaneously measure the expression of selected genes using Real-Time PCR Analysis User Guide PN 68000088 K1. Primers used for qPCR reactions are listed in Additional file 3. Data were analyzed using HTqPCR R package [15] and normalized considering GAPDH, RPS8 and TOP2B as reference genes, as suggested after GeNOrm analysis [16].

### Fatty acid synthase (FASN) enzyme activity

The activity of fatty acid synthase (FASN), a key lipogenic enzyme, was assayed in liver. Tissue samples were first homogenized in 0.25 *M* ice-cold sucrose solution containing EDTA (1 m*M*) and dithiothreitol (DTT, 1 m*M*). Mixtures were ultra-centrifuged at 100,000 x *g* during 1 h at + 4 °C. The resulting supernatants containing cytosolic proteins were collected and frozen at − 80 °C until use. Activity was then assayed on a spectrophotometer at 340 nm absorbance [17] and expressed per unit of cytosolic proteins.

### Functional pathway analysis using GO database

A hypergeometric test was used to select over-represented GO terms for the DEG lists. As a background, we considered all the genes expressed in the analyzed tissue. Our home-made script provided the GO Identifiers, the GO terms, the *p*-value adjusted following Benjamini-Hochberg procedure, the HGNC of genes related to the enriched GO term and their fold-change in expression between diets. The GO terms were considered as over-represented if the corrected *p*-value was < 0.1. Redundant GO terms were finally removed using Revigo [18] and they were manually grouped into representative ancestor terms. An analysis using the webtool DAVID (http://david.abcc.ncifcrf.gov/ [19]) was also carried out and provided similar results.

### Analysis of potential regulatory genes responsible for transcriptome variations

Based on the 2 DEG lists (e.g. down or up regulated by HF diet vs. standard LF diet), potential upstream regulators were defined using different approaches. First, we identified genes encoding a transcription factor or a hormone by looking for GO terms annotations specific to these two types of proteins. Second, we used these DEG lists as inputs for IPA (IPA® - Ingenuity Systems Inc., Redwood City, CA - https://www.ingenuity.com/) to identify potential usptream regulators suggested by this software [20].

### Multiple factor analysis (MFA)

MFA data integration [21] was used to provide a simultaneous view of transcriptomic and metabolomic changes occurring after a diet shift in order to highlight communalities across the two heterogeneous datasets. MFA provides an individual relationship structure by simultaneously evaluating the variation in metabolites and differentially expressed genes (DEGs). When multiple probes matched to one gene, we synthesized the expression data at the gene level by selecting the most varying probe per gene. The MFA method was applied using FactoMiner R package. Briefly, the MFA consists in the following three steps: 1) two principal component analyses (PCA) were performed with the two datasets (here the expression table of the DEG list and the table of metabolite measurements), 2) the components of each PCA were weighed by the first eigenvalue to avoid biasing the MFA towards gene expression (several hundred variables vs. 25 variables for metabolites), 3) the MFA analysis was performed using these weighed variables as new entries, so that expression and metabolite influence was equally distributed into MFA. The first two MFA components (called Dim1 & Dim2) were linked to the components of the two separate PCA by calculating the correlations between MFA and PCA components; in the present study, the two first axis/components of the two PCA were called Dim1_Expr & Dim2_Expr for the expression table and Dim1_Metab & Dim2_Metab for the metabolite table. This allows building a MFA diagnostic plot to study the relationship between the observations, the variables and the tables, facilitating the description of communalities. Calculating the correlations between each trait with the first two MFA dimensions allows deciphering the genes and metabolites mainly responsible for communalities in response to diet shift. A threshold *r* > |0.75| (*p*-value < 0.001) was used to extract these relevant variables. To facilitate the interpretation of the MFA results, diet and line factors were used in the MFA as illustrative variables (they do not contribute to the MFA components construction).

## Results

### Starch substitution with dietary fat and fiber had little effect on growth performance and body composition traits

**Fig. 1:**
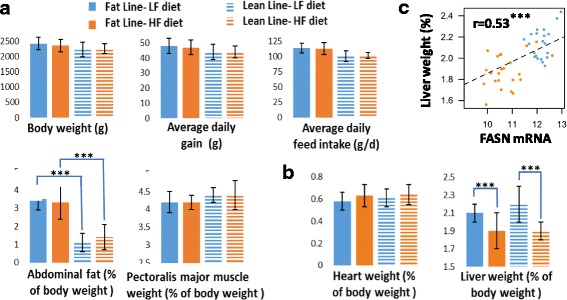
Diet effect on growth performance, body composition and organ weight, Two divergent Fat (solid bars) and Lean (hatched bars) meat-type chicken lines selected for abdominal fat weight, were fed during 6 weeks between 21 and 63 days an iso-caloric diet either enriched in lipids and fiber (HF diet, orange bars) or in starch (LF diet, blue bars). The values are means ± standard deviation with *n* = 12. **a** Growth performance and body composition. **b** Organ weights. **c** Correlation between liver/body weight ration and FASN mRNA level. ***: *p*-value ≤0.001

In the present study, we considered growing chickens originated from two lines divergently selected for abdominal fat weight so that abdominal fat weight represented 3% of total body weight in the fat line and only 1% in the lean line (Fig. 1). Chickens from the two lines were fed either a low-starch, high-fiber, high-fat (HF) diet or a high-starch, low-fiber, low-fat (LF) diet for 6 weeks and were then killed at the same age. The two diets were formulated as isocaloric and isonitrogenous but differed in the dietary energy source available and nutrients.

Performance and body composition at the end of the feeding trial have been fully described in our previous study [3] and are only briefly summarized here (Fig. 1). For both lines, diets had no effects on growth performance and body composition, with the noticeable exception of the liver, which was lighter in birds fed an HF diet vs. an LF diet. Variation in liver weight was significantly correlated (0.53) with the expression of FASN encoding one of the key lipogenic enzymes (Fig. 1c). No interaction between diet and lines was observed for these phenotypes using the present dataset.

### Adaptation to starch substitution was mainly driven by liver transcriptomic changes

**Fig. 2:**
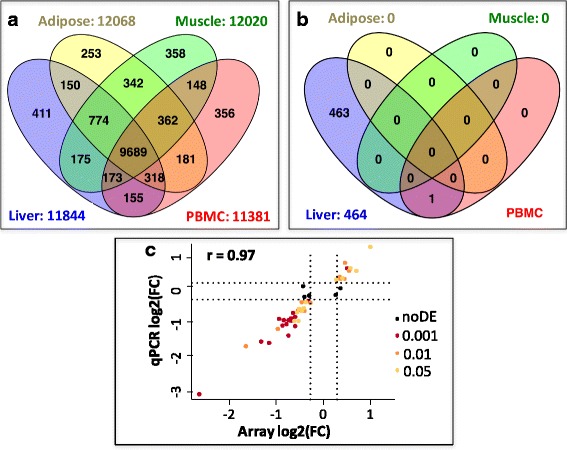
Overview of gene expression and differential expression between diets in liver, adipose tissue, muscle and PBMC. **a.** Number of genes expressed in the 4 tissues. **b** Number of genes differentially expressed between diets in the 4 tissues. **c** Pearson correlation between expression fold change in liver (log2(FC)) for 50 genes analyzed by microarray (x-axis) and RT-qPCR (y-axis) in the same experimental design**;** Significance of the DEG by microarray is indicated by the following color chart: brown dots = *p*-value ≤0.001, orange dots = *p*-value ≤0.01, yellow dots = *p*-value ≤0.05, black dots = node (p.value > 0.05)

To understand the molecular mechanisms leading to the adaptation of birds to changes in dietary energy sources, we analyzed the transcriptomic changes associated with diet in three tissues involved in energy production and use and in circulating blood cells using microarrays. A total of 13,844 genes were expressed in at least one of the four tissues, representing 84% out of the 16,473 genes referenced in the Ensembl v70 annotation, with between 69 and 73% of genes expressed per tissue. Of these expressed genes, 10,870 (65%) were simultaneously expressed in the 3 metabolic tissues (liver, muscle and adipose), and 9689 (58%) were expressed in all 3 tissues and in PBMCs (Fig. 2a). More specifically, 11,844, 12,068, 12,020 and 11,381 genes were expressed in the liver, adipose tissue, muscle and PBMCs, respectively. Among these genes, only a few were tissue specific: 3% on average, with 411, 253, 358 and 355 genes being specific to liver, adipose tissue, muscle and PBMCs, respectively. These results were consistent with previous reports, suggesting that up to 60–70% of protein-coding genes are expressed in any given tissue [5, 12, 22], while very few genes are tissue specific [23–25].

**Fig. 3:**
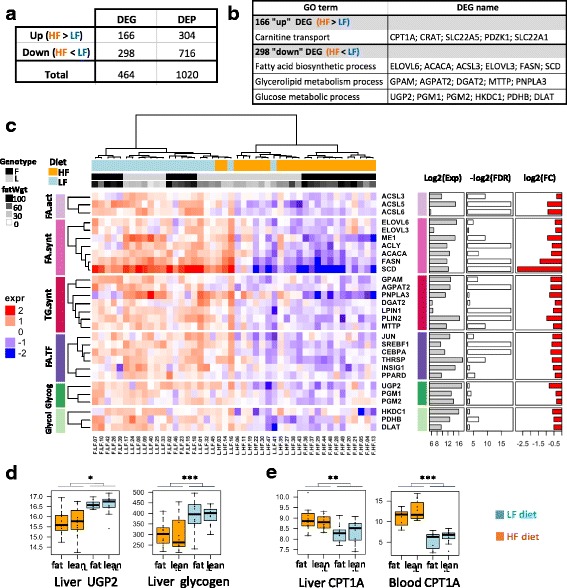
Hepatic expression of key genes involved in fatty acid and glucose metabolism. **a** List of genes up- and down-regulated in HF diet vs. LF diet. DEP: DE probes; DEG: DE genes. **b** GO term enrichment for these two up- and down-regulated gene lists. **c** Heatmap based on gene expression and depicting the main genes related to fatty acid and glucose metabolism. Glycog: glycogen synthesis, Glycol: glycolysis, FA.act: FA activation, FA.synt: FA synthesis, TG.synt: TG synthesis, FA.TF: transcription factor related to FA metabolism. Column annotations: F = fat line L = lean Line, fatWgt = fat weight (g). Row annotations: Exp = mean expression, FDR = corrected *p*-value (False Discovery Rate), FC = fold change between HF and LF diet (ratio HF/LF). **d** Hepatic *UGP2* expression and Glycogen (μmol/g of tissue). **E.** Hepatic and blood *CPT1A* expression

Genotype had major effects in all tissues, with 3341, 3096, 1554 and 6192 DEGs in the liver, adipose tissue, muscle and PBMCs, respectively. No significant interaction between line and diet was observed in any tissues, enabling separate analyses of these two factors. We subsequently focused on the diet effect whereas the genotype effect will be analyzed in another study. Strikingly, regardless of the line, the diet has a significant effect on the transcriptome in the liver only, with 464 DEGs (1020 DEPs) between the HF and LF groups (Fig. 2b and Additional file 4). Among these DEGs**,** 50 genes were further assayed by RT-qPCR, and 45 genes were validated as being differentially expressed between diets (*p* < 0.05) (Additional file 4). We observed that the five invalidated genes (*p* > 0.05, *PPARG*, *ADIPOR2*, *NFE2L1*, *SLC22A4* and *SLC40A1*, black dots in Fig. 2c) had a low expression fold-change between diets, despite the method used. A high correlation (r = 0.97) was observed between the two methods for gene expression quantification (Fig. 2c). Another striking observation was the differential expression of a single gene in PBMCs: *CPT1A*, encoding the carnitine palmitoyltransferase 1, an enzyme involved in the mitochondrial fatty acid β-oxidation (Fig. 3e). All five probes located in either 3’UTR or coding regions show consistent HF/LF expression ratios approximately equal to 2. RT-qPCR assay further confirmed the diet effect on *CPT1A* expression in PBMCs (HF/LF expression = 1.67, *p* = 0.0003).

### Functional characterization of liver transcriptomic changes upon dietary starch substitution

Among the 464 DEGs detected in the liver in response to a change in diet, 298 genes were under-expressed, and 166 genes were over-expressed in HF diet vs. LF diet (Fig. 3a). We next characterized these 2 DEG lists, relying on GO over-representation tests, filtering out GO terms supported by less than 3 genes and considering a *p-*value adjusted with the Benjamini-Hochberg correction ≤5%. These filters suggested functional pathways that were strongly affected by diet (Fig. 3b).

Regarding the 298 down-regulated genes, three GO terms were over-represented, and these genes were related to glucose and fatty acid (FA) metabolism. We therefore performed a finer-grained analysis, focusing on the expression of key regulators and enzymes involved in these pathways (Fig. 3c). Several genes regulating glucose homeostasis were significantly down-regulated in the HF diet vs. the LF diet: HKDC1, encoding an isoform of hexokinase involved in the first step of glucose use in cells; UGP2, PGM1 and PGM2, involved in glycogenesis; and PDHB and DLAT, encoding subunits of pyruvate dehydrogenase, which catalyzes the conversion of pyruvate to acetyl coenzyme A (Fig. 3c). At the metabolite level, we confirmed that glycogenesis was impaired by diet changes, with a significantly lower amount of glycogen in the livers of chickens fed HF vs. LF diets (*p*-value ≤0.0001, Fig. 3d). Additionally, numerous genes encoding key enzymes in FA and TG synthesis pathways were differentially expressed between the diets: ACSL3, ACSL5 and ACSL6, involved in FA activation; ME1, ACLY, ACACA, FASN, SCD, ELOVL6 and ELOVL3, involved in de novo fatty acid synthesis; and GPAT (alias GPAM), AGPAT2, DGAT2, PNPLA3, LPIN1, PLIN2, ACSBG2 and MTTP, involved in TG synthesis, lipid storage, and secretion. We confirmed the down-regulation of most of these genes by HF diet using RT-qPCR (Additional file 4). Among the set of 298 down-regulated genes, the gene most repressed by the HF diet was SCD (7-fold change), which encodes the rate-limiting enzyme of MUFA biosynthesis that converts the saturated fatty acid C18:0 into mono-unsaturated C18:1. Changes in the expression of other genes related to FA and TG synthesis, while significant, were more subtle (1.65-fold change on average, Fig. 3c, right).

Of the 166 up-regulated genes in HF diet vs. LF diet, “carnitine transport” was the only over-represented GO term, with *CPT1A* being the most highly induced gene (logFC = 2). We further observed a significant correlation (r = 0.43, *p*-value ≤0.05) between its expression in the liver and in PBMCs (Fig. 3e), which was confirmed by RT-qPCR (Additional file 4).

### Relationships between hepatic lipid-associated entities observed at the transcriptomic, enzymatic and metabolic levels

**Fig. 4:**
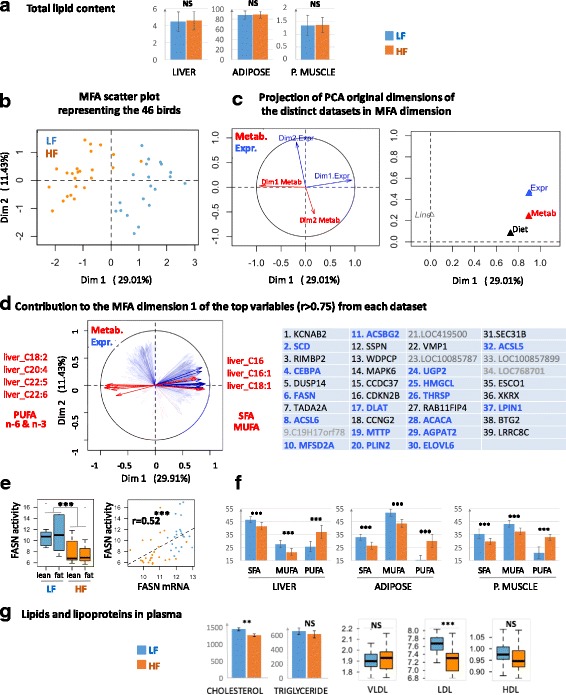
Hepatic fatty acid biosynthesis and secretion was the major metabolism altered in response to the dietary lipid source. **a** Lipid content in three tissues (% of tissue weight). **b**, **c** and **d.** MFA representations related to individuals (**B**) and variables (**C** and **D**). Blue color is related to transcriptomic data and red color to metabolic data. In D, genes in bold blue are known to be involved in FA and TG synthesis storage; grey genes have unknown function. **e** Effect of HF and LF diets on the three FA classes (in % of total FA). SFA: saturated FA (C14:0 + C16:0 + C18:0), MUFA: monounsaturated FA (MUFA (C16:1 + C18:1), PUFA: n-6 and n-3 polyunsaturated FA. *n* = 24 per diet (no effect of lines). **f** Effect of HF and LF diets on the activity of fatty acid synthase (FASN) enzyme and correlation between this activity and the *FASN* expression. *n* = 24 per diet. **g** Plasmatic cholesterol (mg/l), triglyceride (mg/l) and lipoproteins. **: *p*-value ≤0.01, ***: *p*-value ≤0.001, NS: No Significant

The previous analysis highlighted dietary-associated molecular variations in expression levels of genes related to FA synthesis in liver. However, the lipid content in the liver and peripheral tissues, such as muscle and abdominal fat, did not differ between diets (Fig. 4a), suggesting either a disconnection between mRNA level and lipogenic enzymes activities or a genuine repression of lipogenesis concomitant with a direct dietary FA deposition in different tissues when chickens were fed HF diets. Because some fatty acids (e.g.*,* C16:0; C18:1) were more specifically issued from de novo FA synthesis while essential FAs (e.g.*,* C18:2 and C18:3) were derived from dietary lipid deposition, we next integrated the molecular data related to the 465 genes modulated by diet in liver with hepatic FA composition and plasma metabolites using a multiple factor analysis (MFA, see methods). The first dimension of the MFA divided the birds according to diet and explained a high proportion of total variation in traits of interest between the two diets (Fig. 4b). We subsequently determined the variables contributing the most to this dimension. We observed that the first dimension of each PCA performed on each dataset (i.e.*,* transcriptome and metabolites) strongly contributed to the first dimension of MFA (Fig. 4c). We then extracted variables contributing the most to the first MFA dimension (|r| > 0.75, *p*-value< 0.001). Among the 25 metabolites, 7 were selected corresponding to PUFAs in the n-6 and n-3 families (C18:2, C20:4, C22:5 and C22:6) and SFAs and MUFAs (C16:0, C16:1 and C18:1); PUFAs were negative contributors to the first MFA dimension, contrary to MUFAs and SFAs, which contributed collinearly with LF diet (Fig. 4d). Among the 465 DEGs, 39 genes were selected as contributing the most to the first dimension of MFA collinearly with LF diet. Among these genes, five encode unknown proteins, and 17 (50%) are related to FA and TG synthesis or glucose metabolism (Fig. 4d, genes in blue gold). In particular, FASN contributed to the first dimension of MFA and LF diet. Interestingly, we also observed an increase in FASN activity in the livers of chickens fed the HF diet (Fig. 4e left), activity significantly correlated with FASN expression (Fig. 4e right). Conversely, the multiple factor analysis did not reveal genes whose expression changes are collinear with variation of PUFA percentage, suggesting an absence of the transcriptomic regulation of PUFA storage in liver in this situation.

Because de novo synthesis of FA is one of the first steps before lipid secretion and exportation from liver to peripheral tissues, we also studied SFA, MUFA and PUFA proportions in muscle and abdominal adipose tissues. We observed a higher proportion of PUFA in chickens fed the HF diet and higher proportions of SFA and MUFA in the muscle and adipose tissues of those fed LF diets, which paralleled the variations observed in the liver (Fig. 4f). Because there was no transcriptomic change in muscle and adipose tissue associated with diet, these changes in the FA profile were probably related to metabolic rather than transcriptional changes. Consistently, circulating concentration of LDL lipoproteins was significantly increased in plasma of LF diet vs. HF diet, together with plasma cholesterol concentration (Fig. 4g), suggesting a reverse transport of cholesterol to liver after fatty acid uptake by the peripheral tissues.

### Transcription factors and de novo lipogenesis regulation upon starch substitution

**Fig. 5:**
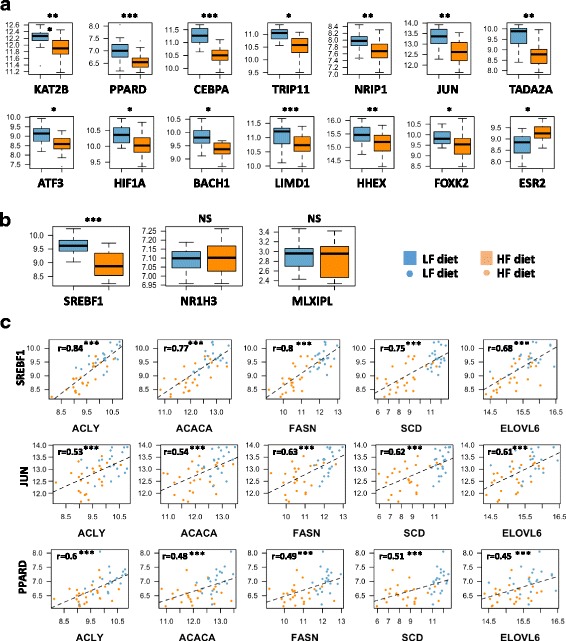
Expression of DE transcription factors, nuclear hormones or transcriptional co-activators in liver of chickens fed HL or LF diets. **a** and **b** Differences in expression levels between HF and LF diets were significant for all genes except *NR1H3* and *MLXIPL*. ***: *p*-value ≤0.001, **: *p*-value ≤0.01, *: *p*-value ≤0.05, NS: No Significant. C. Correlations between SREBF1, JUN or PPARD with different DE genes encoding key lipogenic enzymes

Among the 464 DEGs in the liver, we identified 15 transcription factors, nuclear hormones or transcriptional co-activators (see methods). All of these genes, except ESR2, were down-regulated in HF vs. LF diet, namely, SREBF1 (alias SREBP1), PPARD, CEBPA, TRIP11, TADA2 (alias ADA2a), NRIP1, JUN, ATF3, HIF1A, BACH1, KAT2B (alias P300/CBP or PCAF), LIMD1, HHEX and FOXK2, (Fig. 5a and b). Other transcription factors with well-known roles in lipogenesis regulation, such NR1H3 (alias LXRA) and MLXIPL (alias ChREBP) [26–30], were not found in the list of DEG between HF and LF diets. Using RT-qPCR, we confirmed that NR1H3 and MLXIPL were not DE between the two diets, contrary to SREBF1 (Fig. 5b). Moreover, we observed that SREBF1 expression was highly correlated (*r* > 0.75, *p* ≤ 0.001) to the expression levels of the 3 genes encoding the main enzymes of de novo FA synthesis (e.g.*,* ACACA, FASN and SCD (Fig. 5c)) and to the expression of ACLY, which encodes the ATP citrate lyase, the primary enzyme responsible for the synthesis of cytosolic acetyl-CoA, substrate of ACACA. Notably, significant correlations between ACACA, FASN and SCD gene expression and PPARD or JUN were also observed (0.48 to 0.51 for PPARD, 0.54 to 0.62 for JUN, all with *p* ≤ 0.001 Fig. 5c). However, correlations between lipogenic gene expression and *PPARD* or *JUN* are lower than those observed with *SREBF1*. Finally, the expression levels of NR1H3 and MLXIPL were not significantly correlated with the expression levels of the three genes encoding lipogenic enzymes (e.g., *r* = 0.07 and 0.22 for SCD, 0.02 and 0.3 for FASN and 0.12 and 0.32 for ACACA, respectively).

### Focus on a genomic region involved in the adaptation to starch substitution

**Fig. 6:**
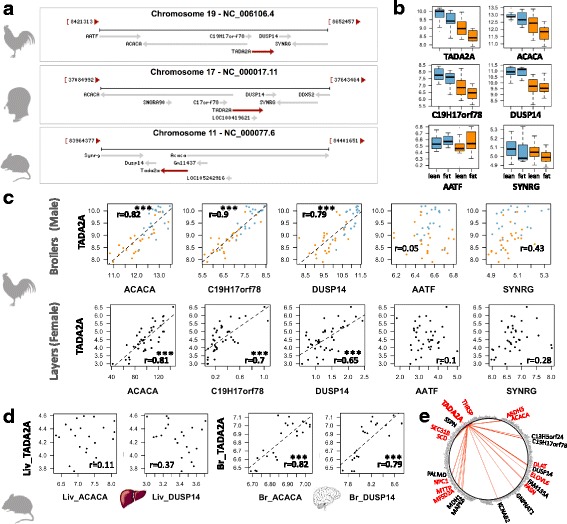
A co-localized and co-expressed gene set containing ACACA and TADA2A. **a** Syntenic region conserved between chicken, human and mouse. **b** Hepatic expression of the syntenic genes in the two diets and genotypes for male broiler chickens. **c** Correlation between TADA2A hepatic expression (taken as reference) and hepatic expression of other co-localized genes in two chicken experimental designs. Top: male broilers analyzed in this study (*n* = 48); bottom: female layers analyzed by RNA-Seq (*n* = 40). Rpkm normalized expression are available in the Additional file 5. **d** Expression in liver (Liv) and brain (Br) of C57BL/6 J mice (*n* = 22) (GDS3232 in GEO profiles [31]. **e** Circle plot depicting the correlation between *TADA2A* and the 298 down-regulated genes in HF diet: red edges indicate genes which expressions are highly correlated to TADA2A expression (*r* > =0.8). Red names indicate genes involved in lipid metabolism. Correlations in bold in the Figure (*r* > 0.7) are highly significant with a *p*-value ≤0.001

For the 464 DEGs, we searched for co-localized genes on the genome, hypothesizing that co-localized genes might be co-regulated. This search revealed a gene cluster composed successively of ACACA, C19H17orf78 (alias C17ORF78 or Gm11437 in mouse), TADA2A and DUSP14 (Fig. 6a) on chromosome 19. These genes were all significantly down-regulated in chickens fed the HF diet (*p*-value ≤0.05) in contrast with more distant genes, such as SYNRG or ATFF (Fig. 6b). Interestingly, the expression of these 4 genes was highly correlated (*r* = 0.82, 0.90 and 0.79, *p*-value< 0.001, between ACACA, C19H17orf78 or DUSP14 and TADA2A taken as a reference because of its central position, Fig. 6c). Such high correlations were also found in another chicken experimental design, corresponding to layers and females (Fig. 6c, Additional file 5). To explore these loci throughout life evolution, we performed a similar analysis on mouse gene expression data from various experimental designs (http://https://www.ncbi.nlm.nih.gov/gds). Expression levels of TADA2A, ACACA and DUSP14 were not correlated in the liver of NMR1 mice fed different diets for 2 weeks (GDS3232 dataset, *n* = 22 from Somel et al. [31]) (Fig. 6d left). However, for the same dataset, the correlation in expression of these genes was significant in the brain (Fig. 6d right). The absence of a correlation between these genes in mice liver was further confirmed in another dataset related to C57BL/6 J mice fed a high-fat diet or a normal diet for up to 24 weeks (GDS6248 dataset, *n* = 51, [32]). Finally, Fig. 6e depicts the genes highly correlated with TADA2A (*r* > = 0.8) among the 298 down-regulated genes in chickens fed the HF diet. We identified 21 correlated genes with significant enrichment in genes related to lipid metabolism: 11 genes among the 21 TADA2A-correlated genes vs. Fifty six genes among the 298 down-regulated genes.

## Discussion

### The liver transcriptome but not the muscle or adipose transcriptome contributed to the adaptation of birds to dietary starch substitution with fat and fiber

The primary objective of this study was to investigate the molecular mechanisms build up by tissues when chickens face changes in their dietary energy source. We first observed that liver weight was impacted by a change in diet, with a significant reduction in chickens fed the HF diet vs. the LF diet. Changes in the liver/body weight ratio have often been reported in response to various stresses and stimuli*,* e.g.*,* in the case of drug administration [33], infectious environment [34], or changes in diet composition [35, 36]. This reduction in liver weight in chickens fed the HF diet may reflect a lower storage of glycogen, as previously observed [36]. Moreover, we also provided new evidence for the overall repression of expression: among the 465 hepatic DEGs, approximately 300 genes were down-regulated in chickens fed the HF vs. LF diets. Finally, whereas a similar number of protein-coding genes were expressed in the four studied tissues, including the muscle, abdominal fat and blood cells, only the liver presented DEGs in response to the dietary changes. Overall, the present study suggests that liver is the keystone organ in metabolic adaptation upon dietary starch substitution with fat and fiber in chicken.

### The main effect of starch substitution with dietary fat and fiber was the reduction in hepatic de novo fatty acid synthesis

The present observations at the transcriptomic, enzymatic and metabolic levels highlight two different types of pathways that were triggered either from the dietary starch or dietary lipids. In both cases, all the action occurred in the liver. In the case of the high-starch LF diet, carbohydrates were used for de novo hepatic FA synthesis, which provides fatty acids that were then secreted from the liver to peripheral tissues through lipoproteins. In the case of the HF diet, dietary fatty acids enriched in n-3 and n-6 PUFA (diet: 56% against 33% of MUFA and 12% of SFA) were directly stored in the different tissues.

These observations, suggesting a direct deposition of FA for chicken fed an HF diet, are further supported by the higher amount of n-3 and n-6 PUFA vs. MUFA+SFA observed in liver, adipose tissue and muscle, since C18:2 and C18:3 cannot be synthesized by animals. Interestingly, no transcriptional adaptation is required for such deposition, since no DEG was observed in adipose tissue or muscle.

**Fig. 7:**
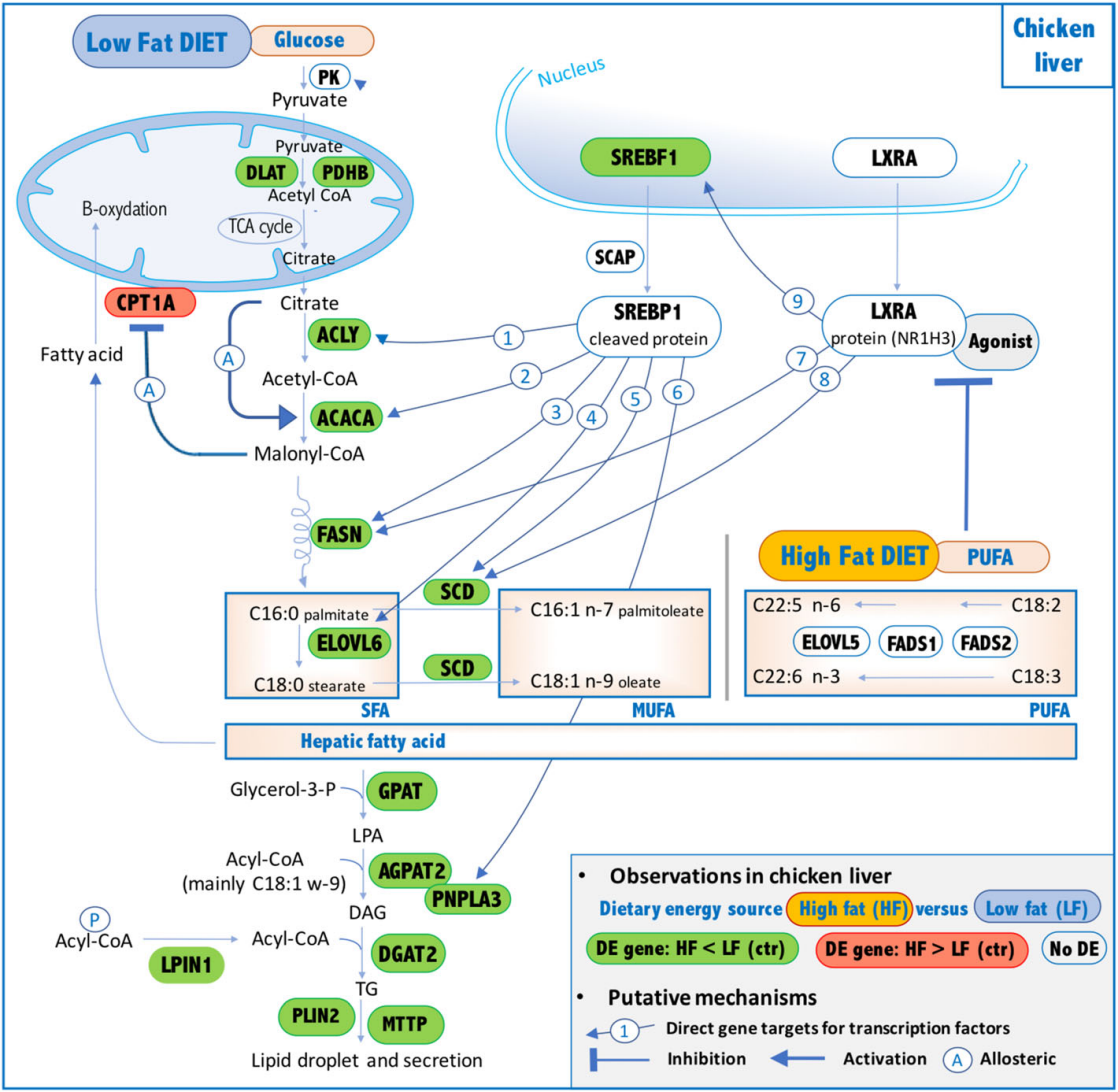
Putative mechanisms explaining hepatic impact of PUFA on genes encoding lipogenic enzymes and SREBF1 and NR1H3 transcription factors in HF diet vs. LF diet. 1- [46, 47]. 2-[48–50]. 3- [51, 52]. 4- [53]. 5- [54, 60, 102]. 6- [60, 102]. 7- [103, 104]. 8- [105]. 9- [106]. 10: [56, 57, 107]

The higher de novo lipogenesis, TG synthesis and export from liver to peripheral tissues in LF were supported at different levels. First, at the metabolic level, we observed an increase in de novo hepatic SFA and MUFA amounts; an increase in SFA and MUFA amounts in muscle and adipose tissue disconnected from any in situ de novo FA synthesis; and finally, an increase in plasmatic low-density lipoproteins (LDL) and total cholesterol, whereas very-low-density lipoprotein particles (VLDL) and total TG were not affected. Indeed, endogenous FA constituting TGs are secreted into the blood in the core of VLDL and then transformed in IDL after liberation of free FA and then in LDL richer in cholesterol. Second, at the enzymatic level, we observed an increase in the hepatic FASN activity. Third, at the transcriptomic level, we highlighted the up-regulation of several genes encoding major enzymes catalyzing steps in the FA synthesis process (i.e., ACACA, FASN, SCD, ELOVL6, ACLY, ME1); FA activation and transport (ACSL3, ACSL5, ACSL6, MFDS2A, FABP7); and TG synthesis, remodeling and packing to VLDL for export to peripheral organs (GPAT1, AGPAT2, LPIN1, DGAT2, PNPLA3, LIPG, PLIN2, MTTP) in the liver of chickens fed the HF diet (Fig. 7). This large number of up-regulated genes explains why GO terms related to FA synthesis and TG synthesis were over-represented in the present analysis, despite the use of *p*-values corrected for multiple testing, which is rare enough to be mentioned. Regarding enzymes involved in TG synthesis, they are characterized by different isoforms (e.g.*,* GPAT1–3, AGPAT1–4 and DGAT1–2). Notably, GPAT1, AGPAT2 and DGAT2, which were shown to be regulated at the transcriptional level in the present study, have been previously reported as the most active isoforms according to a meta-analysis on transgenic mice for these different isoforms [28]. Moreover, in the present study, the top-regulated gene by LF diet encodes SCD, which further supports the well-established pivotal role of this enzyme in FA synthesis and TG secretion. First, SCD catalyzes the conversion of SFA to MUFA (oleyl-CoA) through the initial desaturation of FA. Second, it enhances FA incorporation into TG, since the oleyl-CoA (C18:1 n-9) is the preferential acyl-CoA incorporated in lysophosphatidic acid by DGAT2 at the sn-2 position [37]. As expected, as an upstream pathway of lipogenesis, glycolysis is switched on by the LF diet, as illustrated by the activation of HKDC1, a gene encoding a newly characterized hexokinase isoform [38, 39] catalyzing the first step of glycolysis. PDHB and DLAT genes were also activated, and these genes encode the pyruvate dehydrogenase complex, which provides the substrate linking glycolysis, tricarboxylic acid (TCA) cycle and de novo lipogenesis [40]. This complex has a pivotal position in fueling crosstalk since it catalyzes the mitochondrial conversion of pyruvate – provided by glycolysis – into acetyl-CoA used in TCA cycle as a citrate source. This compound can subsequently exit the TCA and be further converted into acetyl-CoA through a reaction involving ACLY, which was also transcriptionally up-regulated in LF diet (Fig. 7). As expected, dietary carbohydrates provided by the LF diet were used not only in de novo lipogenesis but also in glycogenesis. Indeed, the expression of the genes PGM1, PGM2 and UGP2 – involved in glycogen storage – and hepatic glycogen concentration were increased in chickens fed the LF diet. These transcriptomic and lipidomic observations in chicken liver were consistent with expected changes due to diet composition with less starch in an HF diet; moreover, not only the decrease in glucose derived from starch but also the concomitant increase in dietary lipids in the form of PUFA could be responsible for such changes. Indeed, various studies have reported an inhibition of de novo hepatic FA synthesis by n-3 and/or n-6 PUFA [41–43]. Moreover, among the three metabolic tissues analyzed here, such inhibition occurs solely in the liver, which further emphasizes the established central role this organ plays in lipogenesis in birds [44, 45].

The entire set of genes encoding enzymes involved in de novo lipogenesis was down-regulated in the HF diet. Therefore, we further focused on lipogenic regulators, especially on the three major and well-described hepatic lipogenic transcription factors SREBF1 *(alias* SREBP1*)* [26], NR1H3 (*alias* LXRA) [27, 28] and MLXIPL (*alias* ChREBP) [29, 30]. Among these 3 genes, only SREBF1 was repressed in LF vs. HF diet, and its expression was highly correlated with the expression of genes encoding the key lipogenic enzymes. As previously reported in mammals [46–54], most of the genes encoding enzymes involved in FA and TG synthesis are direct targets of SREBF1 (e.g.*,* ACLY, ACACA, FASN, ELOVL6, SCD, PNPLA3), as illustrated in Fig. 7, which is further corroborated by the present observations. The down-regulation of SREBF1 and lipogenic genes in chickens fed the HF diet is likely related to the dietary PUFAs in the HF diet. Indeed, dietary PUFAs are known to inhibit SREBF1 activity through several mechanisms. First, PUFAs inhibit SREBF1 nuclear uptake (SREBF1 has to be cleaved to enter the nucleus and be active) – even if the mechanism is not yet elucidated; this reduction is mRNA level independent [55]. Second, it has been reported that SREBF1 transcript levels are influenced by PUFA concentration, as observed in the present model [55, 56]. Additional studies further describe a critical role of NR1H3 in the PUFA-mediated down-regulation of SREBF1 mRNA levels [56, 57], indicating that SREBF1 transcripts are inhibited by PUFAs by an antagonizing ligand-dependent activation of *NR1H3*. Nevertheless, there are no reports of a direct effect of PUFA on NR1H3 transcripts so far. The present observations on lipogenic genes and the SREBF1 and NR1H3 transcript regulation observed between HF vs. LF diets support the hypothesis that such regulation may occur in chickens, as summarized in Fig. 7. The well-established ability of dietary PUFAs to decrease the de novo FA synthesis and TG secretion in the livers of mammals was also observed in chicken liver and would involve, at least to some extent, SREBP1 repression, which likely depends on NR1H3 protein. In contrast, the MLXIPL transcription factor does not seem to be involved in the response to PUFAs in the HF diet in chickens. First, the expression of MLXIPL transcript was not decreased in response to the HF diet, while PUFAs suppress MLXIPL activity both by impairing its translocation from the cytosol to the nucleus and by increasing MLXIPL transcript degradation in mammals [30]. Second, MLXIPL plays a pivotal role in the induction of both glycolytic and lipogenic genes by carbohydrate [29, 58]. This is achieved through the binding of this protein to the ChoRE sites in promoters of its target genes involved in either lipogenesis (ACACA, FASN and PNPLA3) [59, 60] or glycolysis (PK) [61, 62]. We did not observe any repression of *PK* transcription, but we did observe a decrease in ACACA, FASN and PNPLA3 transcript levels, likely related to the SREBF1 activity.

The implication of SREBF1 and likely NR1H3 as major transcription factors of de novo lipogenesis regulation does not exclude the putative implication of other transcription factors. For example, PPARD and JUN could play an important role in this regulation. Among the PPAR nuclear receptor family, PPARA and PPARG are well described to activate hepatic FA catabolism as well as adipocytic differentiation and lipid storage. Conversely, PPARD is expressed in various tissues [63] and has multiple but less-characterized functions (for review, [64]. However, several studies reported its role in the regulation of hepatic lipogenesis. Thus, Lee et al. [65] described transcriptional changes occurring in liver, adipocytes and muscle for *db*/*db* mice upon treatment with a PPARD antagonist (GW501516) and highlighted that the hepatic transcriptome was the most responsive, exhibiting an up-regulation of ME1, ACLY, ACACA, FASN, ELOVL6 and GPAT involved in de novo lipogenesis and TG synthesis. More recently, Liu et al. [66, 67] showed that PPARD enhances the use of glucose for glycogen storage and lipogenesis in liver and controls diurnal expression of lipogenic genes in the light-dark/feeding cycle. Altogether, these studies suggest a potential role for PPARD in regulating the differences in lipogenesis we observed between the two diets. JUN represents a potential regulator driving differences in lipogenesis observed in the present study. Indeed, this oncogene is well characterized as playing roles in cell proliferation, cell survival, apoptosis and tumorigenesis [68, 69] and has recently been described as being involved in lipid accumulation by direct binding to SREBF1 promoter (Guo et al. [70]). Interestingly, this study also reports that JUN knockdown in Hep1–6 cells reduced SREBF1 and FASN protein levels and lipid storage. Notably, in the present study, correlations were observed between ACACA, FASN and SCD. Taken together, these results and reports suggest a major role for SREBF1 and potential roles for PPARD and JUN in HF diet-mediated lipogenesis repression.

### *CPT1A mRNA*, the only marker of high-fat diet in blood cells

Focusing on the liver, we noted - upon an HF diet - an activation of *CPT1A* expression, a gene encoding the carnitine palmitoyltransferase 1 [71] that is the β-oxidation rate limiting enzyme for the uptake of long chain fatty acids into the mitochondria. This enzyme ensures the transport across the mitochondrial inner membrane of the long-chain fatty acyl-CoA before β-oxidation. This enzyme is involved in the cross-talk between β-oxidation and lipogenesis, since it is allosterically inhibited by the malonyl-CoA, which is the product of the ACACA activity and the substrate of the de novo lipogenesis. The present results corroborate the key role of *CPT1A* in this cross talk. Strikingly, microarray analysis did not identify any DEG involved in mitochondrial β-oxidation such as *ACADL*, *ACADS* or *EHHADH*, these latter reported as being co-expressed with *CPT1A* in various situations involving β-oxidation such as fasting [12, 42]. Similarly, genes coding peroxisomal β-oxidation enzymes such as *ACOX1–3* that is involved in the PUFA β-oxidation [72], were also not found to be differentially expressed in our model. These last results suggest that the regulation of FA import into mitochondria is the key step for the β-oxidation regulation. In addition, *CPT1A* was the only gene differentially expressed between diets in blood, more precisely in PBMCs. This result is consistent with the observation reported in a recent study using high-fat diet-induced obese rodents [73]. These authors showed an increase in *CPT1A* expression in blood of rats fed an HF diet, further confirmed in the blood of mice fed an HF diet. Another study using pigs fed an high fat-high fiber diet shows similar results [74]. Taken together, these results suggest that *CPT1A* mRNA blood level can be used as a biomarker of high-fat diet in chicken, pigs, rats and mice.

### *TADA2A* and other co-localized genes would be potential new actor of lipogenesis

On chromosome 19, we described a highly conserved cluster of four co-expressed neighboring genes: ACACA, TADA2A, C19H17orf78 and DUSP14. In several species (sheep, rats, mice, cattle and humans), ACACA and TADA2 genes are divergently oriented onto the genome and share a GC-rich bidirectional promoter, which explains their simultaneous expression [75, 76]. Notably, in chickens, four transcripts are described for ACACA in the Ensembl database (Gal-gal 5.0), and a single promoter has been characterized thus far [50, 77], while 23 transcripts and many alternative tissue-specific promoters are described in human genome annotation [78]. In this study, we showed that the expression of these 4 genes located on this genomic locus in the liver is highly correlated in different chicken strains, while such a correlation was not found in mouse liver but was observed in the brain. This latter observation in mice is consistent with those of studies reporting a higher expression of ACACA alternative transcripts derived from the bidirectional promoter shared with the divergently oriented TADA2A gene in mouse brains [75, 78]. While ACACA is a key rate-limiting enzyme involved in FA biosynthesis and plays a pivotal role in cellular energy homeostasis, the biological link between the three other genes and energy regulation is not clear. C19H17orf78 is the ortholog of human C17ORF78 and mouse Gm11437. It was referenced in the Ensembl database V76 (August 2014) and is still referenced in the current NCBI gene database but is absent from Ensembl V87 (Galgal 5.0). The role of this gene is still unknown. DUSP14 belongs to the dual-specificity phosphatase family that includes critical regulators for many biological processes such as T-cell development, immune regulation and tumorigenesis [79, 80]. DUSPs are protein phosphatases catalyzing the dephosphorylation of phosphothreonine, phosphoserine, and phosphotyrosine residues from its own substrates. No association between this gene and hepatic lipids has been reported thus far. TADA2A (alias ADA2A, Transcriptional Adaptor 2A) encodes a subunit of the ATAC (Ada-Two-A-containing) multiprotein complex belonging to the histone acetyl transferase (HAT) complexes, recently renamed the lysine acetyl transferase (KAT) complex. The biological function of KAT complexes is not fully understood, but it plays a crucial role in eukaryotes by regulating chromatin architecture and locus-specific transcription [81–83]. The present study suggests that these three genes—C17ORF78, DUSP14 and, more specifically the gene encoding TADA2A transactivator of the KAT complex—could be involved in hepatic lipogenesis. First, TADA2A shares its promoter with ACACA that encodes a key enzyme from lipogenesis, both being co-expressed in different animal model. Second, the four co-localized genes strongly contributed to dimension 1 of the multifactorial analysis, a dimension that clearly splits de novo-synthesized FAs and dietary PUFAs. Third, the genes most highly correlated with TADA2A (*r* > 0.8) are significantly enriched in lipid-related functions (50% of the 21 genes): ACACA of course but also FASN, SCD and ELOVL6, encoding the 4 key lipogenic enzymes; DLAT, encoding an enzyme that catalyzes the conversion of pyruvate to acetyl-CoA; and MTTP, which is involved in the TG secretion. Interestingly, we found other genes whose expression was highly correlated with the expression of TADA2A and related to lipid metabolism, even if this correlation was not precisely reported for liver so far. MFSD2A is involved in n-3 fatty acid docosahexanoic acid (DHA) transport and in maintaining the integrity of the blood-brain barrier [84, 85]. The role of MFSD2A in hepatocytes remains unclear, even if different reports have shown a role in body lipid metabolism [86, 87]. ABHD5 is a coactivator of PNPLA2 and affects PNPLA3 activity in relation to lipid droplet remodeling [88]. NPC1 is a membrane protein that mediates intracellular sterol transport in late endosomes and lysosomes and participates in cellular cholesterol homeostasis and distribution in different organelles [89]. THRSP (*alias* SPOT14) was discovered three decades ago, and numerous studies have reported a role in de novo lipogenesis. Indeed, the THRSP expression level in a given tissue is correlated with its ability to synthesize lipids (e.g.*,* white adipose tissue, brown adipose tissue, or liver) [90–95]. SPOT14 expression is induced by the thyroid hormone [96] and the constitutive androstane receptor (NR1I3) [97]. More recently, Wu et al. [98] showed that SPOT14 is a direct target of the key lipogenic SREBF1 transcription factor. However, the biochemical mechanism linking SPOT14 to de novo lipogenesis remains unclear. Finally, SEC31B encodes a subunit of the coat protein complex II (COPII) involved in the ER-Golgi transport [99]. Recently, Han et al. showed that SREBF1 is carried from the endoplasmic reticulum to the Golgi in a COPII-dependent manner and then shuttled to the nucleus to induce lipogenic gene expression in response to feeding [100]. According to these results, TADA2A is likely to play a role in the regulation of lipid metabolism. Interestingly, another transcription activator – KAT2B – is also down-regulated in chickens fed an LF diet and belongs to the same HAT/KAT complex family as TADA2A. KAT2N forms a multiprotein KAT complex with KAT2A (alias hGCN5). In addition to acetyl transferase activity toward the histone proteins of these HAT/KAT complexes involving TADA2A or KAT2B, a growing number of non-histone substrates have been identified (for review, see [82, 101]. Taken together, these results and reports suggest a new diet-induced regulatory mechanism for lipid metabolism based on lysine acetylation of histone and/or non-histone proteins through HAT/KAT complex family.

## Conclusions

The present study shows that two different types of pathways are activated depending on the nature of the energy supply, e.g., carbohydrates or lipids. The liver orchestrates this adaptation to lipid-rich diets by a decrease in de novo lipogenesis and TG secretion, through fine-tuned transcriptional regulation. We highlight a hypothesis concerning the potential mechanisms underlying these observations, particularly a likely decisive role of NR1H3/PUFA-mediated SREBF1 repression and the potential implication of other transcription factors, such as PPARD, JUN or TADA2A and KAT2B, in lysine acetyl transferase via the KAT complexes.

## Additional files

Additional file 1: Composition of diets. (XLSX 34 kb)

Additional file 2: Exploration by Principal Component Analysis (PCA) of transcriptomic data (all expressed genes) for each tissue to identify outlier samples. Out of 48, 46, 48 and 44 arrays for liver, adipose, muscle and PBMC respectively, 2, 1, 2 and 1 outlier microarrays were identified by PCA using all the expressed genes. For muscle and PBMC, an additional sample was removed because of an abnormal high number of Agilent-flagged spots or an abnormal background distribution on the array. (PPTX 173 kb)

Additional file 3: Primers used for DEG validation by RT-qPCR. (XLSX 13 kb)

Additional file 4 DEG in liver between the two diets and some features. Array_FC and Array_Pvalue provide the HF/LF expression ratio and the *p*-value adjusted for the multiple test obtained with the microarray technology. qPCR_FC and qPCR_Pvalue provide the HF/LF expression ratio and the *p*-value obtained with the RT-qPCR technology. (XLSX 70 kb)

Additional file 5: Hepatic expression of ACACA, C19H17orf78, DUSP14, AATF and SYNRG for female layers (*n* = 40). RNAseq data are normalized by the classical rpkm method (Chickstress Project – ANR-13-ADAP). (XLSX 54 kb)

## Abbreviations

ACACA: Acetyl-CoA carboxylase
ACLY: ATP citrate lyase
ACSBG2: Acyl-CoA synthetase bubblegum family member 2
ACSL3, ACSL5 and ACSL6: Acyl-CoA synthetases
AGPAT: 1-acylglycerol-3-phosphate O-acyltransferase
ATF: Activating transcription factor 3
BACH1: BTB Domain And CNC Homolog 1
CEBPA: CCAAT/enhancer-binding protein alpha
ChREBP: Carbohydrate response element binding protein
COPII: Coat Complex Component
CPT1A: Carnitine palmitoyltransferase 1A (liver type)
DEG: Differentially expressed gene
DEP: Differentially expressed probe
DGAT: Diacylglycerol acyltransferase
DLAT: Dihydrolipoamide S-Acetyltransferase
DUSP14: Dual specificity phosphatase 14
ELOVL: Elongation of very-long chain
ESR2: Estrogen receptor 2
FA: Fatty acid
FABP: Fatty acid binding protein
FASN: Fatty acid synthase
FOXK2: Forkhead Box K2
GO: Gene ontology
GPAT: Glycerol-3-phosphate acyltransferase
HF: Low-starch high-fat high-fiber diet
HHEX: Hematopoietically expressed homeobox
HIF1A: Hypoxia-inducible factor-1
HKDC1: Hexokinase domain containing 1
JUN: Jun proto-oncogene, AP-1 or transcription factor subunit
KAT2B: Lysine acetyltransferase 2B
LF: High-starch low-fat low-fiber diet
LIMD1: LIM domains containing 1
LPIN1 and PLIN2: Lipin 1 and 2
LXR: Liver X receptor
ME1: Malic enzyme
MFA: Multiple factor analysis
MFSD2A: Major Facilitator superfamily domain containing 2A
MLXIPL: MLX Interacting protein like (alias ChREBP)
MTTP: Microsomal triglyceride transfer protein
MUFA: Monounsaturated fatty acids
NPC1: NPC Intracellular cholesterol transporter 1
NR1H3 (alias LXRA): Nuclear receptor subfamily 1 group H member 3 (alias Liver X Nuclear Receptor alpha
NRIP1: Nuclear receptor interacting protein 1
PCA: Principal component analysis
PCAF: Alias P300/CBP associated factor
PCR: Polymerase chain reaction
PDHB: Pyruvate dehydrogenase (Lipoamide) beta
PGM1 and PGM2: Phosphoglucomutase 1 and 2
PNPLA3: Patatin Like phospholipase domain containing 3
PPAR: Peroxisome proliferator activated receptor
PPARD: Peroxisome proliferator-activated receptor delta
PUFA: Polyunsaturated fatty acids
SCD: Stearoyl-CoA desaturase
SEC31B: SEC31 homolog B
SFA: Saturated fatty acids
SREBF1: Sterol regulatory element binding protein-1
SREBF1: Sterol regulatory element-binding transcription factor 1
TADA2A: Transcriptional adaptor 2A
TG: Triglyceride
THRSP: Thyroid hormone responsive
TRIP11: Thyroid receptor-interacting protein 11
UGP2: UDP-Glucose pyrophosphorylase 2
