# Supplementary figures and images for: Multi-tissue transcriptomic study reveals the main role of liver in the chicken adaptive response to a switch in dietary energy source through the transcriptional regulation of lipogenesis
Source: BMC Genomics. 2018 Mar 7;19:187. doi: 10.1186/s12864-018-4520-5 (PMC5842524; doi:10.1186/s12864-018-4520-5)

## Slide 1
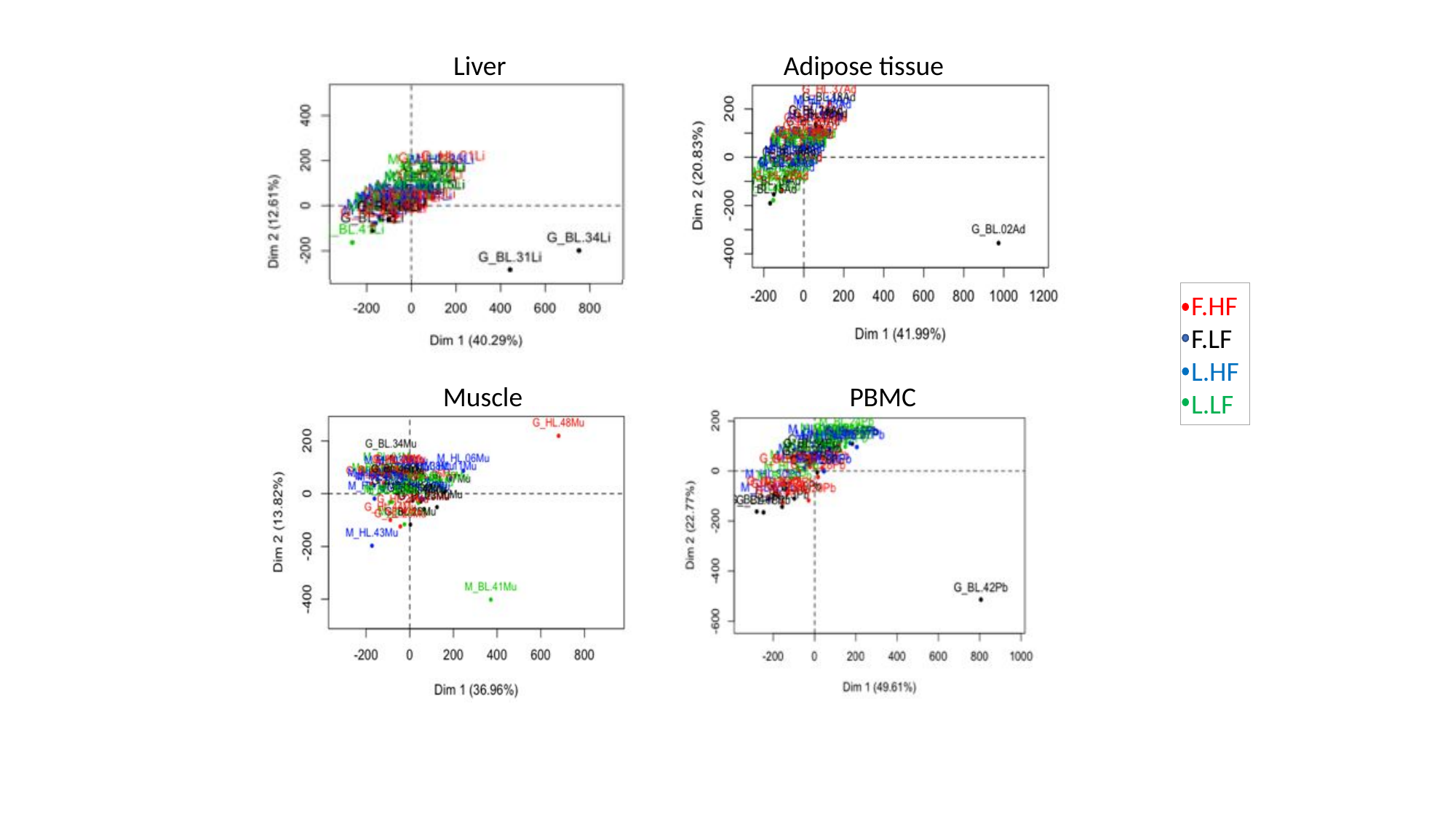

Liver Adipose tissue
F.HF
F.LF
L.HF
L.LF
Muscle PBMC

Supplement: Supplementary file 2 — Exploration by Principal Component Analysis (PCA) of transcriptomic data (all expressed genes) for each tissue to identify outlier samples. Out of 48, 46, 48 and 44 arrays for liver, adipose, muscle and PBMC respectively, 2, 1, 2 and 1 outlier microarrays were identified by PCA using all the expressed genes. For muscle and PBMC, an additional sample was removed because of an abnormal high number of Agilent-flagged spots or an abnormal background distribution on the array. (PPTX 173 kb) [file 12864_2018_4520_MOESM2_ESM.pptx]
